# Human Induced Pluripotent Stem Cell-Derived Mesenchymal Stem Cells Acquire Rejuvenation and Reduced Heterogeneity

**DOI:** 10.3389/fcell.2021.717772

**Published:** 2021-09-16

**Authors:** Wasco Wruck, Nina Graffmann, Lucas-Sebastian Spitzhorn, James Adjaye

**Affiliations:** Medical Faculty, Institute for Stem Cell Research and Regenerative Medicine, Heinrich Heine University Düsseldorf, Düsseldorf, Germany

**Keywords:** heterogeneity, MSC, iMSC, iPSC, rejuvenation, epigenetics

## Abstract

Despite the uniform selection criteria for the isolation of human mesenchymal stem cells (MSCs), considerable heterogeneity exists which reflects the distinct tissue origins and differences between individuals with respect to their genetic background and age. This heterogeneity is manifested by the variabilities seen in the transcriptomes, proteomes, secretomes, and epigenomes of tissue-specific MSCs. Here, we review literature on different aspects of MSC heterogeneity including the role of epigenetics and the impact of MSC heterogeneity on therapies. We then combine this with a meta-analysis of transcriptome data from distinct MSC subpopulations derived from bone marrow, adipose tissue, cruciate, tonsil, kidney, umbilical cord, fetus, and induced pluripotent stem cells derived MSCs (iMSCs). Beyond that, we investigate transcriptome differences between tissue-specific MSCs and pluripotent stem cells. Our meta-analysis of numerous MSC-related data sets revealed markers and associated biological processes characterizing the heterogeneity and the common features of MSCs from various tissues. We found that this heterogeneity is mainly related to the origin of the MSCs and infer that microenvironment and epigenetics are key drivers. The epigenomes of MSCs alter with age and this has a profound impact on their differentiation capabilities. Epigenetic modifications of MSCs are propagated during cell divisions and manifest in differentiated cells, thus contributing to diseased or healthy phenotypes of the respective tissue. An approach used to reduce heterogeneity caused by age- and tissue-related epigenetic and microenvironmental patterns is the iMSC concept: iMSCs are MSCs generated from induced pluripotent stem cells (iPSCs). During iMSC generation epigenetic and chromatin remodeling result in a gene expression pattern associated with rejuvenation thus allowing to overcome age-related shortcomings (e.g., limited differentiation and proliferation capacity). The importance of the iMSC concept is underlined by multiple clinical trials. In conclusion, we propose the use of rejuvenated iMSCs to bypass tissue- and age-related heterogeneity which are associated with native MSCs.

## Introduction

Mesenchymal stem cells (MSCs) (Friedenstein et al., 1970; Caplan, 1991)/medicinal signaling cells (Caplan, 2017)/mesenchymal stromal cells (Horwitz et al., 2005) are multipotent cells with *in vitro* differentiation potential into mesodermal lineages such as adipocytes, chondrocytes, osteocytes, and myocytes. However, per definition *in vitro* cultured MSCs and their mesodermal differentiation potential are not comparable with the *in vivo* case (Keating, 2012; Caplan, 2017). Besides the mesodermal differentiation potential, the definition comprises plastic adherence and positivity for the surface markers cluster of differentiation (CD)73, CD90, and CD105 and negativity for CD45, CD34, CD14 or CD11b, CD79alpha or CD19, and HLA-DR surface molecules (Dominici et al., 2006).

MSCs have shown good therapeutic results in a plethora of studies and clinical trials (Keating, 2012; Jungbluth et al., 2019), yet their characteristics have not been fully clarified or defined. A central question concerns their mechanism(s) of action in therapeutic settings: Is it based on differentiation of MSCs into a target cell type or on paracrine effects triggering surrounding cells to regenerate defective tissue? Evidence exist for both (Boyle et al., 2006; Keating, 2012; Caplan, 2017; Spitzhorn et al., 2018; Jungbluth et al., 2019). Paracrine signaling has been further explored to identify the signaling molecules and exploit their therapeutic effects (Caplan and Correa, 2011; Johnson et al., 2012; Kusuma et al., 2017; Samper Agrelo et al., 2020). The heterogeneity of MSCs and the microenvironment can influence a complex interplay of paracrine and potentially autocrine effects to exert beneficial or detrimental effects, e.g., co-operation of paracrine IL1-signaling and autocrine PGE2 in MSCs and carcinoma cells inducing expression of cytokines, followed by β-catenin signaling and finally formation of cancer stem cells (Li et al., 2012). Like a double-edged sword, MSCs can promote and inhibit cancer (Lee and Hong, 2017; Galland and Stamenkovic, 2020) by various mechanisms depending on their heterogeneity and the exact dosage and timing of the treatment which have to be elucidated further to ensure secure therapies. A better understanding of MSC properties such as immunomodulation, homing to the site of injury and paracrine signaling will give rise to many therapeutic applications, e.g., bi-specific antibody therapy taking advantage of tailored MSCs constantly producing bi-specific antibodies and redirecting T cells to target leukemic cells (Aliperta et al., 2015; Almeida-Porada et al., 2020). Recent publications from the Weinberg lab about epigenetic changes caused by epithelial-mesenchymal transition (EMT) distinguishing cancer stem cells (CSCs) from the non-CSC-tumor cells (Shibue and Weinberg, 2017) and by Carter and Zhao (2020) emphasize the role of epigenetics in cell fate decisions which lead to cellular heterogeneity manifested in distinct lineages and distinct differentiation and disease states.

MSCs are further characterized by predominantly beneficial immunomodulatory properties which is not in the focus of this review but described in more detail, e.g., by Chen et al. (2011); Keating (2012), Wang et al. (2016), and Galland and Stamenkovic (2020) and with the focus on immunotherapy by Almeida-Porada et al. (2020).

MSCs from distinct sources such as bone marrow or adipose tissue differ in certain aspects, e.g., Wagner et al. (2005) found differences regarding mesodermal development and proliferation thus confirming reports of higher proliferative potential of adipose-derived MSCs by Lee et al. (2004). There were several additional studies on heterogeneity in MSCs such as one by Roson-Burgo et al. (2016) and single-cell-sequencing studies (McLeod and Mauck, 2017; Barrett et al., 2019). The single-cell-sequencing technique provides means to assess and possibly tackle intra-population heterogeneity (McLeod and Mauck, 2017; Wolmarans et al., 2021) which can vary in cell culture over time and pose problems to clinical efficacy (Phinney, 2012; Costa et al., 2021; Zha et al., 2021). Heterogeneity is also reflected in a perspective paper by authors from the FDA who regard hematopoietic reconstitution therapies with bone-marrow-derived stem cells as established but see lacking evidence for the clinical efficacy of many other stem-cell based therapies such as adipose-tissue-derived MSC treatments (Marks et al., 2017) thus hinting at a reason for the large discrepancy between the numbers of clinical trials and approval by the FDA. The phenomenon of heterogeneity investigated in all of these studies is related to the global MSC definition providing advantages and disadvantages. Keating discusses the trade-off between a simplistic global definition and potential definitions for MSC subsets (Keating, 2012).

Heterogeneity reflects predisposition for dedicated lineages as proposed by Muraglia et al. (2000) in a hierarchical model for bone marrow MSCs losing lineage potential from osteo-chondroadipogenic via osteo-chondrogenic to osteogenic precursors and excluding osteo-adipo- and chondro-adipogenic lineages. Later, reciprocality of adipogenesis and osteogenesis were reported (Chen Q. et al., 2016). Cell fate commitment of MSCs is transcriptionally regulated by various pathways and this source-associated MSC predisposition may be directed by distinct epigenetic programs (Sui et al., 2020). The nature of MSCs was further elucidated by reports on their perivascular origin in multiple organs (Caplan, 2008; Crisan et al., 2008). Furthermore, the MSC donor age plays an important role in regeneration capabilities which are better in MSCs from young donors. This is a strong argument for MSCs derived from pluripotent stem cells (iMSCs) (Barberi et al., 2005) which meanwhile have been characterized in several studies including (Diederichs and Tuan, 2014; Frobel et al., 2014) and which we found to possess a young or rejuvenated phenotype (Jungbluth et al., 2019; Spitzhorn et al., 2019). Reasons for this age and tissue dependent differences lie in distinct epigenetic programs orchestrating changes in gene expression (Frobel et al., 2014). Carter and Zhao (2020) suggest the use of novel single-cell methods for assaying epigenetic heterogeneity to elucidate processes such as differential priming for cell fate decisions among populations of stem cells.

In this study we investigate the heterogeneity of MSCs from distinct tissues and individuals reflecting distinct genetic backgrounds and ages. We further compare MSCs to MSC populations of distinct tissue origins such as urine-derived renal progenitor cells (UdRPCs; Rahman et al., 2020) and hepatic stellate cells (HSCs; Kordes et al., 2014). Moreover, we explore if the rejuvenation concept manifested in iMSCs can indeed reduce heterogeneity seen in native MSCs.

## Materials and Methods

### Sample Collection for the Meta-Analysis

Samples for the meta-analysis were obtained from NCBI GEO (National center for biotechnology information, Gene expression omnibus) on two levels: first MSC gene expression microarray data from the same platform (Illumina Human HT-12) was downloaded to enable full comparison of expression signals. These datasets are shown in Table 1 and contain the GEO accessions GSE97311 with bone-marrow-derived aged and fetal MSCs and iMSCs (Spitzhorn et al., 2019), GSE149171 with adipose-tissue-derived MSCs (Jung et al., 2020), GSE77227 with kidney-derived perivascular stromal cells (Leuning et al., 2017), GSE77272 with tonsil-, bone- marrow-, and adipose-tissue-derived MSCs (Park et al., 2016), GSE59662 with anterior-cruciate-derived MSCs (Lee et al., 2015), and GSE52841 with Wharton’s jelly-derived MSCs (Sukarieh et al., 2014). Second, datasets of multiple technical platforms were downloaded for binary assessment if genes were expressed thus enabling comparison of symbols related to expressed genes. These datasets are shown in Table 2 and contain the GEO accessions GSE128281 with urine-derived renal progenitor cells (UdRPCs; Rahman et al., 2020), GSE100448 with amniotic-fluid-derived MSCs (Spitzhorn et al., 2017), and GSE152250 with hepatic stellate cells (Zhang et al., 2020).

**TABLE 1:** Samples on platform Illumina Human HT-12.

GSE97311_samples	GSE77272_samples	GSE52841_samples	GSE59662_samples	GSE77227_samples	GSE149171_samples
Spitzhorn et al. (2017)	Park et al. (2016)	Sukarieh et al. (2014)	Lee et al. (2015)	Leuning et al. (2017)	Jung et al. (2020)
Pubmedid:30885246	Pubmedid:27224250	Pubmedid:25129543	Pubmedid:25729860	Pubmedid:28191776	Pubmedid:32961
aged_MSC_74y	adipose_tissue_MSC_1	UC_SGA_MSC_1	total_knee_arthroplasty_MSC_1	bmMSC1	adipose_MSC_siControl_1
iPSC_MSC_74y_viral	adipose_tissue_MSC_2	UC_MSC_1	total_knee_arthroplasty_MSC_2	bmMSC2	adipose_MSC_siControl_2
iMSC_74y_viral	adipose_tissue_MSC_3	UC_SGA_MSC_2	anterior_cruciate_ligament_MSC_1	bmMSC3	adipose_MSC_siControl_3
aged_MSC_62y	adipose_tissue_MSC_4	UC_SGA_MSC_3	anterior_cruciate_ligament_MSC_2	kPSC1	adipose_MSC_siDGCR8_1
iPSC_MSC_62y_episomal	bone_marrow_MSC_1	UC_MSC_2	anterior_cruciate_ligament_MSC_3	kPSC2	adipose_MSC_siDGCR8_2
fetal_MSC_3	bone_marrow_MSC_2	UC_MSC_3	anterior_cruciate_ligament_MSC_4	kPSC3	adipose_MSC_siDGCR8_3
iPSC_MSC_fetal_line_1_viral	bone_marrow_MSC_3	UC_MSC_4			adipose_MSC_Early_pasage_1
iPSC_MSC_fetal_line_1_episomal_1	bone_marrow_MSC_4	UC_SGA_MSC_4			adipose_MSC_Early_pasage_2
iPSC_MSC_fetal_line_1_episomal_2	tonsil_MSC_1				adipose_MSC_Early_pasage_3
iMSC_fetal_line_1_viral	tonsil_MSC_2				adipose_MSC_Late_passage_1
iMSC_hESC_H1	tonsil_MSC_3				adipose_MSC_Late_passage_2
H1_a	tonsil_MSC_4				adipose_MSC_Late_passage_3
H1_b				groups:	
H9_a		UC: umbilical_cord		bone_marrow_MSC	
aged_MSC_60y		SGA: Small for Gestational Age		kidney perivascular stromal cell	
aged_MSC_70y					
fetal_MSC_1					
fetal_MSC_2					
groups:					
bone_marrow_MSC					
fetal_MSC					
iMSC					
ESC					
iPSC					

**TABLE 2:** Samples of amniotic-fluid-derived MSCs (AF-MSCs), urine-derived progenitor cells (UdRPCs), and hepatic stellate cells (HSCs).

GSE100448_samples	GSE128281_samples	GSE152250_samples
Spitzhorn et al. (2017)	Rahman et al. (2020)	Zhang et al. (2020)
Affymetrix Human Primeview	Affymetrix Human Primeview	Illumina HiSeq 4000
Pubmedid:29225627	Pubmedid:31959818	Pubmedid:33205063
AF_MSC_1	UdRPC_UM54	HSC_US-1564046
AF_MSC_2	UdRPC_UM48	HSC_US-1564053
	UdRPC_UF60	HSC_US-1564057
	UdRPC_UM27	HSC_US-1564058
	UdRPC_UF61	HSC_US-1564061
	UdRPC_UM51	HSC_US-1564063
	UdRPC_UF45	HSC_US-1564067
	UdRPC_UF31	HSC_US-1564072
	UdRPC_UF21	HSC_US-1564073
		HSC_US-1564080
		HSC_US-1564088
		HSC_US-1564090
		HSC_US-1564091
		HSC_US-1564092
		HSC_US-1564098
		HSC_US-1564117
		HSC_US-1564127
		HSC_US-1564136

*AF, amniotic fluid; UdRPC, urine-derived renal progenitor cell; HSC, hepatic stellate cell.*

### Meta-Analysis of Datasets on the Illumina Gene Expression Platform

Datasets in form of non-normalized data listed in Table 1 were downloaded from NCBI GEO and imported into the R/Bioconductor (Gentleman et al., 2004) using the package *lumi* (Du et al., 2008). Detection-*p*-values were available for all datasets except for the dataset with accession no. GSE149171 with adipose-tissue-derived MSCs (Jung et al., 2020). Detection-*p*-values for this dataset were generated by fitting a loess model to detection-*p*-values and logarithmic (base 2) expression values of the comparable dataset with accession no. GSE52841 and applying this model to predict the detection *p*-value from the logarithmic expression values of dataset GSE149171. A threshold of det-*p*-value < 0.05 was employed to judge if a gene was expressed. Imported Illumina data was transformed to the log2 scale and normalized via quantile normalization. Hierarchical clustering analysis for the generation of the dendrogram was performed with the method *hclust* using Pearson correlation as similarity measure and complete linkage as agglomeration method and the package dendextend (Galili, 2015) for the color bars indicating the cell types. Heatmaps were generated employing the method *heatmap.2* from the R package gplots (Warnes et al., 2015).

### Extension of the Meta-Analysis by Datasets on Diverse Transcriptome Platforms

Datasets in form of Affymetrix raw data (CEL files) and summarized Illumina next-generation sequencing (NGS) data listed in Table 2 were downloaded from NCBI GEO and imported into the R/Bioconductor (Gentleman et al., 2004) using the package *affy* (Gautier et al., 2004) for the datasets with accession nos. GSE128281 and GSE100448 on the Affymetrix Human Primeview platform. Detection-*p*-values were calculated as described in our previous publication (Graffmann et al., 2016). A detection-*p*-value < 0.05 was employed to identify expressed genes. For the NGS dataset GSE152250 with hepatic stellate cells a threshold of FPKM > 1 was applied to filter expressed genes. ENSEMBL gene ids were mapped to gene symbols using the annotations from ENSEMBL Biomart version 103 (Haider et al., 2009).

### Venn Diagrams and Gene Ontology Analysis

Venn diagrams were employed to dissect subsets of genes expressed at detection-*p*-value < 0.05 (microarrays) or FPKM > 1 (NGS data). Venn diagrams were generated with the R package *gplots* (Warnes et al., 2015). Subsets of genes expressed exclusively in distinct MSC types were further subjected to over-representation analysis employing the Bioconductor package GOstats (Falcon and Gentleman, 2007). The most significant GO-terms were plotted as bar charts via the R package ggplot2 (Wickham, 2009) of the negative logarithm (base 10) of the *p*-values indicating the number of involved genes and the ratio of involved genes to the total of genes in the GO-term on a color scale.

## Results

### Comparison of MSCs of Different Sources in a Meta-Analysis

**FIGURE 1:**
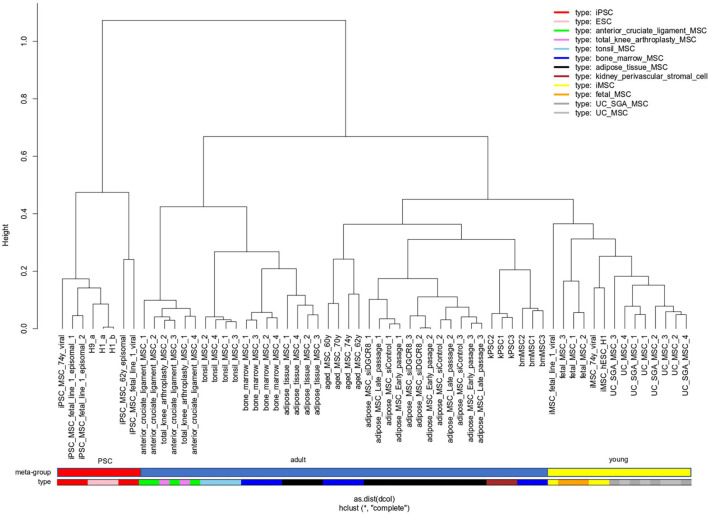
“Young” MSCs cluster separated from adult MSCs and PSCs. Pluripotent stem cells (PSCs, red color bar) of embryonic or reprogrammed (iPSCs) origin cluster separated from MSCs. Within the MSCs one cluster contains all samples related to “young” origin: fetal MSCs, umbilical cord (UC) MSCs and iMSCs (derived from iPSCs), marked with a yellow color bar. Adult MSCs (blue color bar) of various origins are spread over the remaining clusters.

In a meta-analysis of transcriptome data, we compared MSCs of distinct sources which includes bone marrow (BM), adipose tissue (AT), umbilical cord (UC), cruciate, tonsil, kidney, and iMSCs listed in Table 1 and also Pluripotent stem cells (PSCs). With respect to pluripotency and age, we classify samples into three meta-groups *PSC*, *young*, and *adult*. Embryonic stem cells (ESCs) and induced pluripotent stem cells (iPSCs) fall into the meta-group PSC, UC-derived, and fetal MSCs into the meta-group *young* as well as iMSCs for which we had shown the rejuvenated phenotype in a previous publication (Spitzhorn et al., 2019). The remaining samples are classified as *adult*. Figure 1 shows that *young* MSCs (yellow color bar) cluster separately from *adult* MSCs (blue color bar). However, in a superior cluster, young and adult MSCs are joined and cluster separately from PSCs (red color bar) of embryonic or iPSC origin. This grouping of samples reflects the differences between pluripotent and multipotent cells as manifested in their transcriptome. Adult and young MSCs fall into the same group of multipotency but are distinguishable between each other pertaining to age-related heterogeneity within MSCs. Interestingly, kidney perivascular stromal cells cluster with adult MSCs thus confirming the common nature of MSCs and pericytes as suggested by Caplan (2008) based on the study by Crisan et al. (2008).

### Common and Distinct Gene Expression Signatures

**FIGURE 2:**
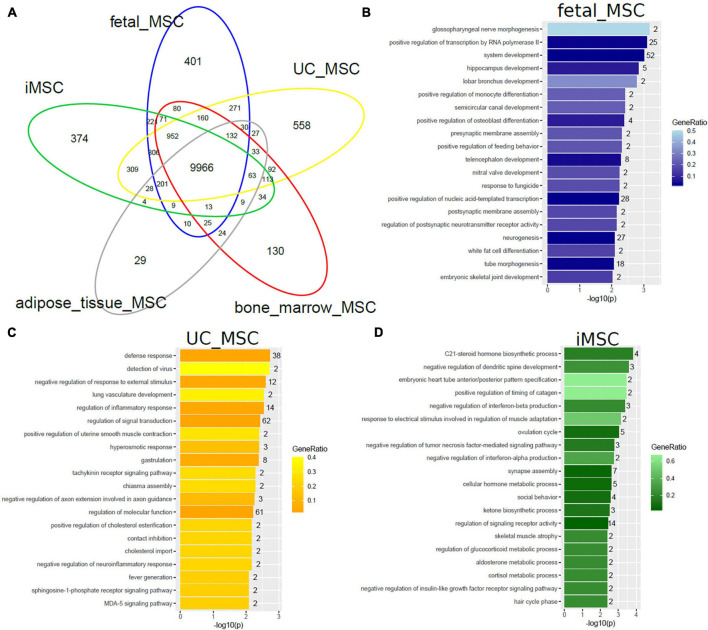
MSCs of distinct origin share a large common gene signature but differ in smaller specific gene signatures. **(A)** The Venn diagram comparing gene expression in five MSC datasets on the Illumina Human HT-12 platform shows that most (9966) genes are expressed (detection-*p*-value < 0.05) in common. Specific to distinct types are subsets containing smaller numbers of genes: fetal MSCs-401, iMSCs-374, adipose-tissue-MSCs-29, bone-marrow-MSCs-130, and UC-MSCs-558. Over-representation analysis of Gene ontologies (GOs) in the specific subsets revealed GO terms of which the 20 most significant are listed here. **(B)** Significant GO terms in fetal MSCs relate to developmental and morphogenetic processes. **(C)** Significant GO terms in UC-MSCs relate to defense/inflammatory response, lung development, gastrulation. **(D)** Significant GO terms in iMSCs relate to hormone metabolism, neuronal development, interferon, and TNF signaling.

We set out to further investigate heterogeneity between distinct MSC types by comparing gene expression (detection-*p*-value < 0.05) on the same technical Illumina Human HT-12 microarray platform. The venn diagram in Figure 2A shows that MSCs of distinct origin share a large common gene signature of 9966 genes but differ in tissue-type specific gene signatures (Supplementary Table 1A). The two most significantly over-represented GO terms (Biological Process) in the common 9966-gene signature (Supplementary Table 1G) are *primary metabolic process* (*p* = 6E-108) and *cellular macromolecule localization* (*p* = 1.1E-96).

**FIGURE 3:**
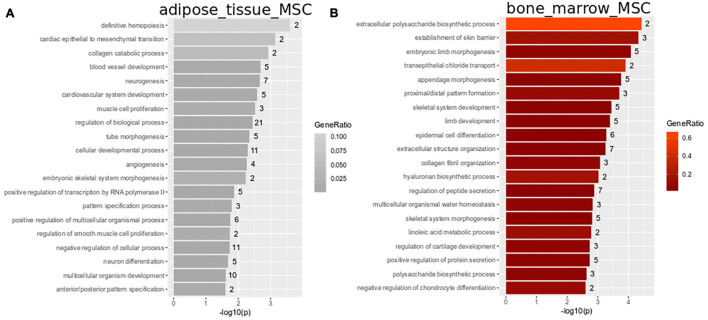
Heterogeneity in adipose-tissue and bone-marrow-derived MSCs reflects tissue of origin. Subsets of genes exclusively expressed in adipose tissue-derived MSCs (29 genes in Figure 2A) and bone-marrow-derived MSCs (130 genes in **A**) were analyzed for over-representation of GO terms. **(A)** Most significantly over-represented GO terms in adipose tissue-derived MSCs include vascular, muscle and neuronal development which may be considered characteristic for adipose tissue. **(B)** Most significantly over-represented GO terms in bone-marrow-derived MSCs include skeletal system and limb development pointing at osteogenic properties.

Subsets of the venn diagram are dissected to distinct types containing smaller numbers of genes: fetal MSCs-401, iMSCs-374, adipose-tissue-MSCs-29, bone-marrow-MSCs-130, and UC-MSCs-558. Over-representation analysis of Gene ontologies (GOs) in the specific subsets revealed GO terms of which the 20 most significant are listed here. These GO terms reflect the heterogeneity characterized by the specific original microenvironment of the MSCs: significant GO terms in fetal MSCs relate to developmental and morphogenetic processes (Figure 2B and Supplementary Table 1B), significant GO terms in UC-MSCs relate to defense/inflammatory response, lung development, gastrulation (Figure 2C and Supplementary Table 1C) and significant GO terms in iMSCs relate to hormone metabolism, neuronal development, interferon, and TNF signaling (Figure 2D and Supplementary Table 1D). While these MSCs are associated with the young, developmental phenotype, Figure 3 demonstrates that the most significant GO terms in adult MSCs, in adipose-tissue and bone-marrow-derived MSCs also reflect the tissue of origin. The subsets of genes exclusively expressed in adipose tissue-derived MSCs (29 genes in Figure 3A and Supplementary Table 1E) and bone-marrow-derived MSCs (130 genes in Figure 3B and Supplementary Table 1F) were analyzed here. Figure 3A reveals that the most significantly over-represented GO terms in adipose tissue-derived MSCs include vascular, muscle, and neuronal development which may be considered characteristic for adipose tissue. Figure 3B reveals that the most significantly over-represented GO terms in bone-marrow-derived MSCs include skeletal system and limb development pointing at osteogenic properties.

### Tissue of Origin Determines Heterogeneity of Amniotic-Fluid-Derived MSCs, Urine-Derived Renal Progenitor Cells and Hepatic Stellate Cells

**FIGURE 4:**
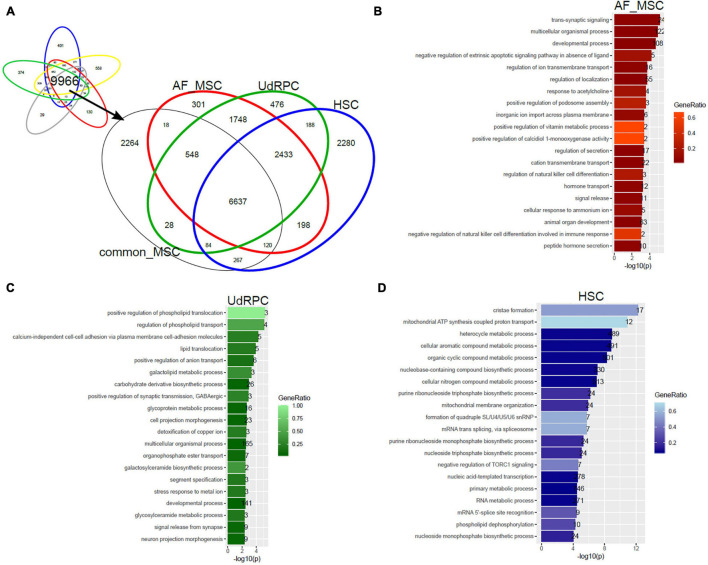
Tissue of origin determines heterogeneity of amniotic-fluid-derived MSCs (AF-MSCs), urine-derived renal progenitor cells (UdRPCs), and hepatic stellate cells (HSCs). **(A)** Genes expressed in common in MSCs on the Illumina platform (9966 genes) were compared with AF-MSCs, UdRPCs, and HSCs. Subsets of genes exclusively expressed in AF-MSCs (301 genes in **A**),UdRPCs (476), and HSCs (2280) were analyzed for over-representation of GO terms. **(B)** Most significantly over-represented GO terms in AF-MSCs include developmental and signaling processes which may be considered characteristic for embryonic development. **(C)** Most significantly over-represented GO terms in UdRPCs include lipid transport and detoxification pointing at renal properties. **(D)** Most significantly over-represented GO terms in HSCs include metabolic, cristae and mitochondrial processes pointing characterizing liver.

In a follow-up analysis we aimed at extending the transcriptome comparison of MSCs to MSC populations of distinct tissue origins measured on different microarray platforms and by RNAseq. Figure 4 shows this comparison with additionally amniotic-fluid-derived MSCs (AF-MSCs), urine-derived renal progenitor cells (UdRPCs), and hepatic stellate cells (HSCs). We characterized UdRPCs in a previous publication (Rahman et al., 2020), these SIX2-positive renal progenitor cells meet the criteria defined for MSCs. HSCs are activated upon injury and inflammation in the liver leading to fibrosis (Gressner and Weiskirchen, 2006; Wruck et al., 2017; Driscoll and Patel, 2019). Furthermore, an immune-modulatory beneficial effect of MSCs on HSCs has been described (Parekkadan et al., 2007; Driscoll and Patel, 2019). Beyond that, HSCs themselves have been characterized as MSCs with the ability to reduce liver fibrosis (Kordes et al., 2014). We used the genes expressed in common in MSCs on the Illumina platform (9966 genes, Figure 2A) and compared them with genes expressed in AF-MSCs, UdRPCs, and HSCs (Figure 4A). The resulting subsets of genes exclusively expressed in AF-MSCs (301 genes in Figure 4A), UdRPCs (476), and HSCs (2280) were analyzed for over-representation of GO terms (Supplementary Table 2). The most significantly over-represented GO terms in AF-MSCs include developmental and signaling processes which may be considered characteristic for embryonic development (Figure 4B), the most significantly over-represented GO terms in UdRPCs include lipid transport and detoxification pointing at renal properties (Figure 4C) and the most significantly over-represented GO terms in HSCs include metabolic, cristae, and mitochondrial processes pointing at liver characteristics (Figure 4D).

### Characteristics of MSCs Derived From Induced Pluripotent Stem Cells

**FIGURE 5:**
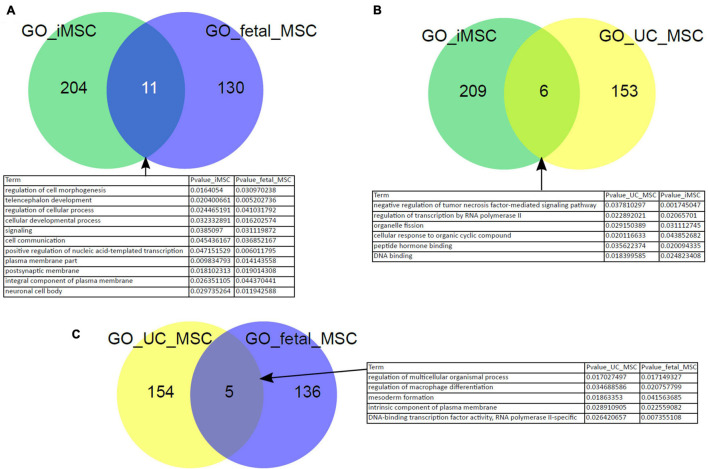
Characteristics of iMSCs overlap with those of MSCs from young origin. Overlapping over-represented GO terms between iMSCs, fetal MSCs, and UC-MSCs were identified. The overlap is connected to developmental processes **(A–C)**.

In our previous work we have reprogrammed fetal MSCs to iPSCs and then differentiated these back into MSCs, so called iMSCs. These iMSCs exhibited the typical MSC characteristics such as plastic adherence, spindle-shaped morphology, expression of cell surface markers CD73, CD90, and CD105 as well as PDGFRβ by parallel absence of hematopoietic and pluripotency markers such as OCT4. These iMSCs had a similar pattern of secreted molecules as native MSCs and showed a typical MSC-like differentiation capacity into adipogenic, chondrogenic, and osteogenic lineage *in vitro* (Spitzhorn et al., 2018, 2019). In addition, we previously showed that iMSCs are reset to a rejuvenated phenotype with a much reduced heterogeneity compared to native tissue-specific MSCs. To gain further insights and confirmation of similarities between iMSCs and native MSCs, we investigated the Biological processes present in the GO terms over-represented in the exclusive subsets of *young* MSC types iMSCs, fetal MSCs, and UC-MSCs from the venn diagram in Figure 2A. Figure 5 shows that there are overlapping over-represented GO terms of iMSCs with those of MSCs of young origin. The overlap is associated with developmental processes (Figures 5A–C). Thus, the heterogeneity between iMSCs, fetal MSCs, and UC-MSCs manifested in distinct expressed genes eventually leads to common developmental biological processes.

The process of MSC rejuvenation refers to a process in which the cells are reversed into a more embryonic state or the suppression of aging-inducing processes. Besides the generation of iMSCs, other methods have been described to induce this rejuvenation process in MSCs. MSCs can be either completely reprogrammed to iPSC or can be partially reprogrammed. These processes lead to changes in DNA methylation, histone composition, and epigenetic patterns. An alternative approach is the treatment of the cells with specific microRNAs leading to a rejuvenated stage. A further strategy for inducing rejuvenation events is management of reactive oxygen species (ROS levels). Several substances such as ascorbic acid or lactoferrin have been described to be able to reduce ROS levels and delay cellular senescence. Another relevant feature in aged cells is declining mitochondria function. Thus, several approaches target to improve mitochondria function in aged cells by upregulation of key proteins such as HSPA1L or FGF21. Also, the specific over-expression of key transcription factors such as FOXP1, YAP, or FOXD1 support the rejuvenation process. Another angle for attenuating aging is related with autophagy management. Inhibition of pathways such as mTOR can increase autophagy mechanisms and as such support protein homeostasis which is diminished in aged cells (Zhou et al., 2020). A recently described method for rejuvenation of human MSCs employs extra cellular vesicles collected from infant MSCs which are used to treat MSCs from elderly persons. In response to this treatment downregulation of ROS production in the elderly cells was observed and furthermore a better functionality regarding their ability in decreasing necrotic areas in diabetic mouse models was observed (Khanh et al., 2020).

### Cluster Analysis With Aging-Rejuvenation- and Epigenetics-Related Gene Signatures

**FIGURE 6:**
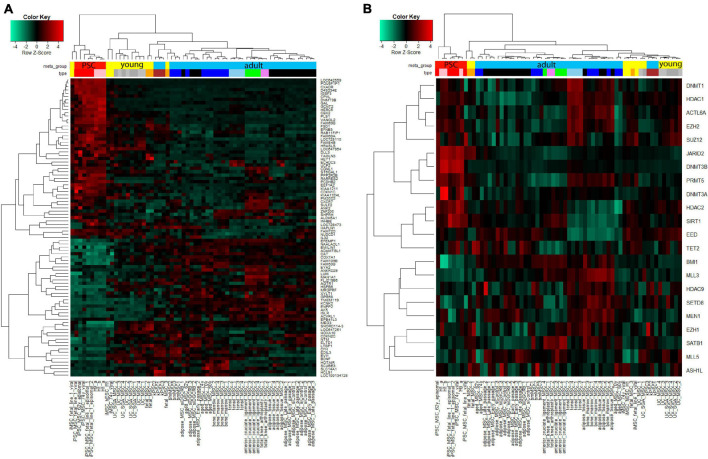
Cluster analysis displays agglomeration of young (iMSCs, fetal, and UC-MSCs) MSCs in epigenetics-related gene signatures. **(A)** Heatmap using the aged-rejuvenation gene signature from our previous publication (Spitzhorn et al., 2017) yields coherent clusters of PSCs and iMSCs with fetal and UC-MSCs. **(B)** Also the heatmap using the epigenetics-related gene signature from the publication by Avgustinova and Benitah (2016) yields coherent clusters of PSCs and iMSCs with fetal and UC-MSCs, however, a bit more fragmented than in **(A)**.

We further aimed at identifying factors contributing toward the heterogeneity phenotype typical of MSCs and their reversion to the rejuvenated state associated with iMSCs. Figure 6A shows cluster analysis and heatmap using a gene signature which we found to be associated with aging and rejuvenation of MSCs at the transcriptome level in our previous publication (Spitzhorn et al., 2019). The analysis resulted in distinct clusters of PSCs and iMSCs with fetal and UC-MSCs. In Figure 6B we retrieved an epigenetics-related gene signature from the publication by Avgustinova and Benitah (2016) and could demonstrate distinct clusters of PSCs and iMSCs with fetal and UC-MSCs which, however, were a bit more fragmented than in Figure 6A.

### Role of Epigenetics in Heterogeneity of MSCs

Epigenetic modifications are crucial for determining the gene expression profile of a given cell type. They are defined as mechanisms which enable or prevent access of the transcription machinery to genomic loci in a heritable and at the same time flexible manner (Bernstein et al., 2007). The most commonly analyzed factors in this regard are DNA methylation (DNAme) which occurs at chromosomal regions where CpG dinucleotides are overrepresented (Gardiner-Garden and Frommer, 1987) and histone modifications (Strahl and Allis, 2000). Promoter associated DNAme suppresses gene expression while histone modifications act as activating or repressing factors depending on the context and the nature of the modification (Bernstein et al., 2007).

Epigenetic factors have been described to regulate MSC heterogeneity in two distinct ways: (1) tissue- specific gene expression patterns and (2) age-specific gene expression patterns (Avgustinova and Benitah, 2016; Sui et al., 2020). Together, these two layers define the differentiation and regeneration potential of MSCs.

Most analyses of the epigenetic landscape of MSCs have been carried out by comparing BM and AT MSCs, which have distinct differentiation potentials (Schellenberg et al., 2011; Xu et al., 2017). While BM MSCs readily differentiate into the osteogenic lineage, this potential is reduced in AT MSCs, which favor the adipogenic differentiation. DNAme analysis of the promoters of key transcription factors such as *runt-related transcription factor 2 (RUNX2*) and *peroxisome proliferator activated receptor*γ (*PPAR*γ) revealed an opposing profile with *RUNX2* being demethylated in BM MSCs and methylated in AT MSCs while *PPAR*γ was unmethylated in these cells and methylated in BM MSCs (Xu et al., 2017). Similar results were obtained when including MSCs derived from other tissues (Reinisch et al., 2015). Interestingly, only BM derived MSCs differentiate efficiently into chondrocytes and are capable of building hematopoietic niches after transplantation which has been related to their special gene expression and epigenetic profile (Reinisch et al., 2015).

Gene ontology analysis of the differentially methylated regions in BM vs. AT MSCs revealed that they correlate with the distinct functions of the cells similarly to the results we obtained when comparing the gene expression profiles of various MSCs (Figure 1; Schellenberg et al., 2011). Interestingly, the differential methylation of only 2 specific CpG sites reliably distinguishes between MSCs derived from BM and from AT (de Almeida et al., 2016).

The equilibrium between opposed differentiation pathways and self-renewal is not only mediated by DNAme but also by histone modifications. It has been shown that high levels of the Polycomb complex 2 factor Enhancer of Zeste 2 (EZH2) favor adipogenesis of BM MSCs (Hemming et al., 2014; Jing et al., 2016), while its antagonist lysine demethylase 6A (Kdm6a), as well as SET-domain-containing 2 (Setd2) mediated H3K36me3 and Ashl1 mediated H3K4me3 are necessary to maintain the osteogenic differentiation potential (Wang et al., 2018; Yin et al., 2019).

When we analyzed the expression level of a panel of epigenetic factors described by Avgustinova and Benitah (2016) in our data set, we saw a good clustering according to tissue type as well as age (Figure 6B).

With aging, the differentiation potential of BM MSCs changes from osteogenic to more adipogenic which is associated with impaired bone regeneration and a higher risk for osteoporosis (Chen Y.-H. et al., 2016; Ganguly et al., 2017). This is related to an increase in EZH2 and H3K27me3 levels (Chen Y.-H. et al., 2016). In addition, the DNAme profile of MSCs changes profoundly during aging. In BM MSCs regions with repressive chromatin marks tend to gain DNAme comparable to observations made in blood cells, while open regions marked by H3K4me1 loose DNAme during aging (Fernández et al., 2015).

To overcome age-induced impairments of MSCs, we and others have derived MSCs from iPSCs, so called iMSCs which have a rejuvenated transcriptome and epigenetic profile (Figures 6A,B; Frobel et al., 2014; Spitzhorn et al., 2019) and thus promise to be particularly suitable for regenerative therapies.

Regarding the therapeutical application of MSCs, a third layer of heterogeneity is added by cell culture induced replicative senescence. MSCs do not all have the same proliferation and differentiation capacity *in vitro* and over time the highly proliferative fibroblastoid colony forming units decline continuously from initially 20% to 1% after 2 months of cultivation (Schellenberg et al., 2011). Based on their DNA methylome, cells can be clearly separated according to their tissue of origin even after several weeks of culture. However, focusing only on senescence-associated genes, the tissue differences recede behind the senescence associate differences (Schellenberg et al., 2011). This is not due to chromosomal aberrations but to DNAme changes which were significantly enriched in regions with repressive histone modifications, namely H3K9me3, H3K27me3 as well as EZH2/Polycomb target regions (Schellenberg et al., 2011), which is similar to the observations made during aging of MSCs.

MSC function is crucial for tissue homeostasis and in this regard their heterogeneity presents advantages as well as disadvantages. On the one hand, a defined tissue related transcription profile points to highly adapted cells that are perfectly prepared for the requirements of the given tissue. On the other hand, aging-related heterogeneity shows that the transcriptional control gets impaired over time. This does not only reduce the capability of MSCs to maintain homeostasis but also is a risk factor for disease development as the propagation of a defective transcriptional program during cell division of MSCs increases the risk of developing malignancies (Sui et al., 2020).

### Impact of MSC Heterogeneity in Pre-clinical and Clinical Trials

#### Application of MSCs in Clinical Trials

Initially, MSCs were described to be supportive in the generation of new blood cells (hematopoiesis) (Maximow, 1924). Because of their immunomodulatory capacity, MSCs are widely used in the treatment of graft versus Host disease (GvHD; Le Blanc et al., 2008). They have a multilineage differentiation potential as manifested in their ability to evolve into cells from the mesodermal lineage such as bone, cartilage, and fat (Dominici et al., 2006; Crisan et al., 2008; Fitzsimmons et al., 2018). Furthermore, various reports attest that MSCs are also able to form other cell types *in vitro* or *in vivo*, such as pancreatic β cells, cardiomyocytes, or liver cells (Segers and Lee, 2008; Cho et al., 2018; Spitzhorn et al., 2018). MSCs create a special microenvironment by secreting chemokines, growth factors, extracellular vesicles, and cytokines with immunomodulatory effect and additionally residential tissue specific progenitor cells are guided to the area of regeneration/injury (Galipeau and Sensébé, 2018; Harrell et al., 2019; Rodríguez-Fuentes et al., 2021). Their secretory profile allows MSCs to support regeneration activities by the release of for example HGF, VEGF-A, PDGA, and a plethora of interleukins (Fitzsimmons et al., 2018). By sensing and reacting to environmental factors MSCs can influence the immune system by supporting T-helper 2 cells response. In addition, MSCs can influence the process of antigen-presentation and can decrease expansion and recognition of T-cells (Caplan, 2017). A big advantage in the use of MSCs for cellular therapies are their low expression levels of human antigen class II Molecules, thus allogenic transplantation of cells without HLA matching is possible (Bloor et al., 2020).

All these characteristics make MSCs very promising for the use in innovative therapeutic approaches. In line of this, MSCs are or have been used in over 1,000 clinical trials for versatile applications in the various organs and diseases.^1^ Neurological and orthopedic (joint) impairments, cardiovascular disease, and GvHD are the top four indications for MSC applications (Kabat et al., 2020). China and United States are the main drivers of MSC-based trials (Rodríguez-Fuentes et al., 2021). By the year 2020 there have been more than 300 completed clinical trials using MSCs (Levy et al., 2020). Reacting to the current pandemic situation, first studies using MSCs in the treatment of COVID-19 have been started and show their broad potential use cases (Leng et al., 2020; Shetty, 2020).

In total, MSC therapies have been shown to have an acceptable safety profile with parallel beneficial therapeutic effects in several clinical studies. The number of clinics offering stem cells therapy is constantly increasing; so is the number of companies working on MSC related products. But his also comes along with mal-practice and misuse of those cells (Zhou et al., 2021). Although many clinical trials have shown the safety of transplanted MSCs, the beneficial outcome is often not clearly shown. In contrast to the immense research undertaken in MSC-based clinical trials, only a small number of MSC-based products or therapies have been approved to date. MSCs were successfully used in pioneer studies for cellular therapies in the human system in 1995, where they were autologously transplanted to support the hematological recovery (Lazarus et al., 1995). Their immunosuppressive activity is frequently exploited in transplantation settings to diminish the risk of organ rejection or graft-versus-host-disease (GvHD; Fitzsimmons et al., 2018). A phase 3 clinical trial where MSCs are used for treatment of complex perianal fistulas in Crohn’s Disease (Panés et al., 2016) is one of the most successful latest trials (NCT01541579). Although the mode of action is not fully proven but indicated to be related to immunomodulatory capacity, this MSCs based product, Alofisel, was approved by the European Medicines Agency in 2018 (Levy et al., 2020). Over the course of time ten MSC products have been approved (numbers of products in brackets) for use in fistulas in Crohn’s disease (2), GvHD (2), subcutaneous tissue defects (1), amyotrophic lateral sclerosis (1), knee cartilage defects (1), spinal cord injury (1), critical limb ischemia (1), and acute myocardial infarction (1). Thereof one product is approved in Europe, one in Canada and New Zealand, one in Japan, five in South Korea, and one in India (Levy et al., 2020). We refer to Table 1 from the publication of Levy et al. (2020) for a summary of the application, administration routes and clinical efficacy of MSCs in clinical trials for different diseases. Levy et al. (2020) – referring to Galipeau and Sensébé (2018) – report a typical dose of 1–2 million MSCs/kg for intravenous injection into humans.

Most MSC-based clinical trials use adult autologous or allogeneic MSCs from the iliac crest (bone marrow), placenta, or adipose tissue (Rodríguez-Fuentes et al., 2021). Allogeneic MSC transplantation is evaluated to be the most promising route due to larger possible scales and use of less invasive procedures. With compliance to GMP standards this possibility is a safe, accessible treatment option for the patients (Rodríguez-Fuentes et al., 2021) although native MSCs are rare and only accessible by complex processes *in vivo* (MacQueen et al., 2013). The usual route of MSC administration is intravenous transfusion followed by local injection (Rodríguez-Fuentes et al., 2021). The most effectives doses were calculated to be around 150 × 10^6^ cells per patient (Kabat et al., 2020), in line with the 1–2 million cells/kg suggested by Galipeau and Sensébé (2018).

In addition to the before mentioned use cases, other abilities of MSCs are under investigation. Since MSCs can evade the host immune system partially, MSCs have been employed as drug delivery vehicles for example in anti-cancer treatment since they are also able to home into tumor tissue (Sasportas et al., 2009). Many of these MSC-based cancer treatments have reached the clinical stage now (Levy et al., 2020). In contrast to other cellular therapeutic agents, MSCs strategies do not only rely on cell contact or differentiation effects but also on their paracrine effector function (Levy et al., 2020). Since a large number of studies is only relying on the released molecules, the cells direct regenerative and differentiation potential is not necessarily in the focus of such investigations (Wilson et al., 2019). This is why the concept of “medicinal signaling cells” has been introduced (Caplan, 2017). Exploiting MSC secreted factors or extracellular vesicles is of high potential but the engineering and scaling process of such released factors have to be established in a standardized manner to enable broad clinical use (Phinney and Pittenger, 2017).

#### MSC Heterogeneity Is a Multi-Level Issue Which Influences the Outcome of Clinical Trials

The general problem of clinical trials is the long certification process which leads to the fact that the final end-therapeutic agent is 5–10 years behind the latest research advances. Although over 1,000 clinical trials using MSCs have been performed, many MSC therapies fail in the later clinical stages, which is reflected by the fact that so far only ten MSC-based products have reached (partial) market approval. One of the main reasons for this are the many layers of heterogeneity associated with the use of MSCs: inter-cellular differences and various cell (sub)populations, inter-tissue differences and inter-donor or inter-recipient variations. Another layer of heterogeneity is introduced by the researchers themselves by the use of different protocols for cryopreservation, up-scaling and administration (Tanavde et al., 2015; Levy et al., 2020).

#### Cells/Tissues

A big hurdle which needs to be overcome to finally have a broad success in MSC-based therapy is the cells’ intrinsic heterogeneity. It is well described that MSCs are a heterogeneous cell population (O’Connor, 2019) and is a big challenge for researchers to develop robust therapy strategies (Zha et al., 2021). Cellular heterogeneity has impacted the effectiveness of MSC therapies in animal models and has been cited as a possible factor contributing to the variability in treatment outcomes of MSC therapies in clinical trials (O’Connor, 2019). First of all the tissue and the donor (age, health status) dictate MSC quality. The difference in tissue-specific MSCs may result from the present varying microenvironments (niches). MSCs preparation from distinct tissues have shown a certain degree of heterogeneity as revealed in the meta-analysis included in this study and by whole transcriptome data and single cell RNA sequencing (Huang et al., 2019; Liu et al., 2019; Sun et al., 2020). Clinical efficacy is also influenced by the fact that MSCs from distinct tissue sources also exhibit variation in their differentiation lineage potential (Zhou et al., 2021). Not only inter-tissue differences have been shown but even amongst sampling sites of the same tissue, the same niche has led to different outcomes (Costa et al., 2021). One key finding in MSC-based therapeutic use it that there is a transfer gap between the surface marker expression which is associated with *in vitro* function and the corresponding *in vivo* effect (Wilson et al., 2019). Depending on the tissue MSCs are derived from they exhibit varying immunophenotype and differentiation potential. An example for this is the finding that MSCs from the dental pulp predominantly differentiate into neuronal cells (Suchanek et al., 2009) whereas MSCs from birth-associated tissues such as amnion and umbilical cord have a high prevalence to evolve in hematopoietic direction (Sivasubramaniyan et al., 2012). Bone marrow derived MSCs have a great differentiation capacity into osteogenic and chondrogenic direction (Vinardell et al., 2012), whereas MSCs from adipose tissue are more prone for adipogenic differentiation (Lotfy et al., 2014). This is why, in addition to the classical MSCs markers other MSC subpopulations are under investigations CD271+, CD49f+, CD146+, and Stro-1+ (Zha et al., 2021). Addressing molecular heterogeneity amongst various MSC preparations is necessary to ensure a high degree in efficiency and safety of MSC therapies and will support assessment of proper cell quality control, scaling strategies, and application therapeutical strategies (O’Connor, 2019).

#### Variabilities Between Donors

In addition to this inter-cellular multiplicity, it has been shown that potency and the quality of MSCs has huge inter-donor variation (Xin et al., 2010). The function of MSCs is influenced by donor parameters such as age, health status, sex, and the region the MSCs are derived from Zha et al. (2021). From the donor side, MSC quality is hampered for example in disease conditions such as diabetes, rheumatoid arthritis, or Parkinson’s disease (Costa et al., 2021). For example it was shown that MSCs derived from the bone marrow of patients suffering from osteoarthritis had a lower multi-lineage differentiation potential (especially into cartilage direction) and an altered cell surface marker pattern than from healthy donors (Čamernik et al., 2020). Precisely in the immunosuppressive actions of MSCs, significant inter-donor variation have been reported (Ketterl et al., 2015). Although MSCs have been used in many clinical trials for immune-related diseases with promising results, the individual immune reaction can be very heterogeneous (Carvalho et al., 2019). These differences in the host immune reaction in relation to cellular therapies are an important factor (Levy et al., 2020). Many studies have underlined the variable proliferation and differentiation capacity as well as their secretory profile and their anti-tumor effect when emanating from distinct tissues or donors (Costa et al., 2021). The typical clinical scenario is dominated by elderly patients. For these people, the potential of their MSCs is diminished because of cellular aging-associated effects such as senescence, genome instability, oxidative stress, and DNA damage (Zhou et al., 2008; Reuter et al., 2010; Moskalev et al., 2013; Yang et al., 2018). All these aging-related characteristics negatively influence the differentiation capacity and functionality and thereby the ultimate therapeutic performance (Oreffo et al., 1998; Stolzing and Scutt, 2006; Wagner et al., 2009).

#### Handling of MSCs in Culture

The described levels of MSC heterogeneity are multiplied by differences in the various researcher- triggered parameters in the *in vitro* stages prior to admission (Madsen et al., 2017). MSCs derived from primary tissue sources are restricted in their use since long term culture is influencing their proliferation and differential potential as well as their phenotype (Wagner et al., 2008). Clinical trials require large numbers of MSCs, this results in the need of prolonged *in vitro* expansion which results in early senescence and altered gene expression patterns which have a negative effect on the therapeutic potential (Li et al., 2020). The cell isolation processes, culture conditions such as medium, substrates, and O_2_ concentration, have significant influence on the characteristics of MSCs as well as cryopreservation and thawing routines are critical steps influencing quality. The culture conditions have to mimic the specific stem cell environment. MSCs can be cultured as clones, however, heterogeneity was observed to be inter- and intra-clonal (Costa et al., 2021; Zhou et al., 2021). Despite the cellular variations, another very important factor is the handling of the MSCs including, expansion, freezing, thawing, and administration processes since all these steps can have significant influence on the therapeutic outcome (Levy et al., 2020). Handling factors such as injection volume, needle, injection site, buffer used, all have different physical properties such as shear stress and all contribute toward heterogeneity (O’Cearbhaill et al., 2014). All of these MSC culture condition and parameters have been shown to influence global cellular signatures and therefore need to be critically investigated for optimal design of the scaling process for subsequent clinical success (O’Connor, 2019).

#### Strategies to Overcome These Challenges

At first the *in vivo* study management in pre-clinical studies has to be improved to enable more successful clinical trials. Especially in the orthopedic field mostly small animal models are used and therefore the human scenario is insufficiently reflected. The big gap from pre-clinical to clinical stage is partially caused by the fact that (small) animal models require a much lower cell number as compared to the human system where prolonged *in vitro* culture of MSCs are needed (Wilson et al., 2019).

Although there are many promising results in MSC-based trials, other trials were discontinued after disapproval by the FDA due to poor quality of the controls, and heterogeneity of MSCs with regard to stability, differentiation, and migratory potential (Conrad et al., 2009; Haga et al., 2015). Other drawbacks in the use of primary MSCs is the logistical and financial complex process of continual donor identification, qualification, and recruitment. Furthermore, large cell quantities are needed for cellular therapy which makes it necessary to culture the initially obtained cells *in vitro* which can have also negative impacts such as reduced stemness (Bloor et al., 2020). Clinical results have already indicated that MSCs which have undergone minimal expansion prior to transplantation perform better in the case of GvHD (von Bahr et al., 2012). In contrast to normal biological and chemical-based drugs, MSC-based therapies are dynamic and more complex (Levy et al., 2020). Biological drugs require a clear identification, purity and the efficacy of the product. The active substance in the cellular product is clear to name. Although strategies are there to increase cell purity, authorities so far do not necessarily insist to have a completely pure product. There seems to be a general acceptance of heterogeneity within a cellular product which makes the whole landscape of MSC-based clinical trials very complex. Nevertheless, the most important aspect is to define the exact mode of action of the cellular agent including the upstream and downstream molecular events (Wilson et al., 2019). Furthermore, MSC subpopulation identification and understanding as well as mastering the robust standardized manufacturing process of MSCs are important aspects for the establishment of more MSC-based therapies (Levy et al., 2020; Zha et al., 2021). Many MSC based clinical trials are company driven. This is associated with lack of fully disclosure of all study relevant information with regard to intellectual property protection which makes is very difficult to compare the different trials with each other (Kabat et al., 2020). One route to overcome certain levels of this inconsistent trial outcomes is to set relevant quality parameters for MSCs. The initial MSC minimal criteria for the ISCT were focusing on the stemness ability of MSCs (Levy et al., 2020). The recent position statement on nomenclature also include the tissue origin and the functional capacity which have major influences on the mode of action (Viswanathan et al., 2019). The development of artificial intelligence strategies can support unraveling the so far undetermined factors within MSC-based therapies (Zhou et al., 2021). Prior assessment of MSCs surface marker composition as well as gene signature may advance the prediction in therapy outcome and may lead to more effective cell manufacturing processes (Lipsitz et al., 2016). The distinct cell populations applied in the different studies are rarely completely characterized which makes it very challenging to compare the various studies and furthermore to reproduce them (Wilson et al., 2019). Approaches for the development of new protocols for stable MSC propagation *in vitro* to enable larger yields or the establishment of large MSC banks are promising but need critical evaluation (Lechanteur et al., 2016; Wilson et al., 2019). Potency assays should be carried out to address MSC functionality, the currently accepted assay is the suppression of *in vitro* T-cell proliferation (de Wolf et al., 2017).

Several biomaterial approaches have been developed with the aim to increase the homogeneity of MSCs during the propagation phase prior to clinical use (Levy et al., 2020). For example, specific biological materials can be used to improve MSCs delivery and survival (Zhou et al., 2021). An example for this is the loading of MSCs with microparticles which encapsulate small-molecules (Tzouanas et al., 2014) or synthetic polymers as ECMs. Furthermore, gene therapy approaches may lead to improved MSC performance for a particular application and could increase the degree of homogeneity. There is a clinical trial underway to investigate the use of genetically modified MSCs in treating Kabuki Syndrome (NCT03855631) (Zhou et al., 2021). Another way to support MSC treatment efficiency is the priming of MSCs before admission. With this approach, MSCs were shown to be in an exogenously boosted more potent state compared with the unprimed cells (Zhou et al., 2021). Going away from transplanting cells toward transplanting secreted factors such as extracellular vesicles has also shown promising results in trials for GvHD or chronic kidney disease (Lai et al., 2018; Dreyer et al., 2020). Engineering processes for large scale production of MSC derived extracellular vesicles could be a standardized process (Zhou et al., 2021).

### iMSCs as a Complementary Cell Source for Therapy

#### Pre-clinical Use of iMSCs

As described above, there are many substantial issues in the use of native MSCs for therapeutic approaches. Because of these issues, MSCs so far have only been successfully converted into treatment of patients to a certain extent. In contrast to native MSCs with all their restrictions, an unlimited and safe source such as iPSC-derived iMSCs would be a great alternative (Zhao and Ikeya, 2018). Although the collection processes of for example bone marrow and the subsequent isolation of MSCs are standardized and carried out in a routine fashion, it is still an invasive process and is associated with donor-site morbidities (Bhumiratana et al., 2016). Furthermore, the cell number for transplantation is a critical point. Since the initial cell number from bone marrow aspirates is restricted, cells have to be expanded *in vitro* which is associated with the problem of decreased proliferation and differentiation potential upon prolonged *in vitro* culture (Duscher et al., 2014; Palumbo et al., 2014; Yang et al., 2018). This challenge also could be overcome by the use of iMSCs since iPSCs can be indefinitely expanded and then differentiated in the needed amount of iMSCs. By their ability to indefinite self-renewal iPSCs are similar to ESCs (Prigione et al., 2011; Drews et al., 2012). The iPSCs can be differentiated into MSCs, the so called iMSCs. These iMSCs show similar characteristics as native MSCs with regard to their immunophenotype, differentiation potential, and secretory profile (Chen et al., 2012; Frobel et al., 2014; Kimbrel et al., 2014; Hu et al., 2015; Spitzhorn et al., 2019). As alternative to the use of primary MSCs the route via iPSCs into iMSCs is promising. Upon the reprogramming process into iPSCs, the cells are shifted into a phenotype which does not show characteristics of an aged cell. Additionally, there are reports that iMSCs are in a rejuvenated state compared to the primary MSCs. Reports have shown that iMSC lose the age-related and tissue-specific DNA methylation profiles but maintain a donor specific DNA methylation signature (Lian et al., 2010). Especially the use of iMSCs from iPSCs which were derived from stem cells which are obtained non-invasively such as urine are a very promising alternative (Rahman et al., 2020). When the initial cell material is chosen wisely, iPSCs can be obtained with non- or minimal invasive procedures and they are ethical unproblematic. When used autologous or with matching HLA type the frequency of unwanted immune reactions is diminished (de Rham and Villard, 2014; Bohndorf et al., 2017). Several protocols have been developed to generate iMSCs from iPSCs including the use of FGF supplementation or the inhibition of TGFb signaling (Zhao and Ikeya, 2018). As mentioned above most MSCs are derived from bone marrow or other tissues. Since invasive procedures are necessary, the donor is faced with several risks (Sheyn et al., 2016). Since iPSCs can be expanded limitless, this broad available starting material has the potential to increase the homogeneity and standardized procedure in iMSC use.

iMSCs have been used in various *in vivo* models for multiple sclerosis, limb ischemia, autoimmunity, hypoxic ischemia and autosomal inherited liver disease and bone defect healing (Lian et al., 2010; Gruenloh et al., 2011; Kimbrel et al., 2014; Wang et al., 2014; Hawkins et al., 2018; Spitzhorn et al., 2018; Jungbluth et al., 2019). Interestingly, it has been reported that iMSCs were even able to outperform their native counterparts (Wang et al., 2014; Hawkins et al., 2018), particularly concerning the release of exosomes and support of mitochondrial transfer function (Li et al., 2014; Zhao and Ikeya, 2018). Zhang et al. (2016) and Yang et al. (2020) report mitochondrial transfer in iMSCs co-cultured with rodent cellular models of induced neuronal and cardiac injury. The mitochondrial transfer is accomplished by nanotubules. Zhang et al. (2016) suggest that higher expression of Rho GTPase 1 (MIRO1) and TNFaIP2 leads to superior mitochondrial transfer in iMSCs compared to BM-MSCs via tumor-necrosis-factor-alpha-(TNF)-induced tunneling nanotube formation. Although they are derived from potential tumorigenic pluripotent cells, none of the previous mentioned studies have reported tumor formation of the iMSCs and in a liver regeneration study they did not cause any signs of tumor formation after 2 months (Spitzhorn et al., 2018). The generation and propagation of iPSC-derived MSCs was shown to be applicable to GMP conditions in a routine manner (Ozay et al., 2019). Thawed iMSCs had a low degree of senescence and were immunomodulatory active by IDO mediated immune suppression (Chinnadurai et al., 2017).

#### Clinical Trials Employing iMSCs

Another re-assuring evidence in support of the use of the iMSC concept in regenerative medicine and cell replacement therapy is their use in clinical trials. Up until now, iMSCs have been implemented in six clinical trials. Cynata Therapeutics Ltd. (Australia) has finished a phase I clinical trial investigating the safety, tolerability and efficacy using iMSCs which were derived from iPSCs treating steroid resistant acute GvHD in adults with preliminary promising results (NCT02923375). The company furthermore is just recruiting patients for a study to examine the early efficacy of intravenous administration of iMSCs derived from iPSCs in adults which have been brought to an intensive care unit with COVID-19 infection (NCT04537351). In a second phase of this study the effect of iMSCs on the Acute Respiratory Distress Syndrome will be assessed. The company has now announced a Phase 3 clinical trial of CYP-004, its Cymerus mesenchymal stem cell (MSC) product for osteoarthritis. Another study which is performed by the Tongji Hospital (China) in cooperation with the Chinese Academy of Sciences is investigating the safety of ESC-derived iMSCs in treating meniscus injury patients in a phase I clinical trial (NCT03839238). The Department of Urology of the Asan Medical Center in Seoul, South Korea is running a Phase 1 Study to investigate the safety of human ESC-derived iMSCs in the treatment of Interstitial Cystitis (NCT04610359). Another Phase 1 safety study is carried out by The First Affiliated Hospital of Zhengzhou University in China. Aim of this study is to evaluate the safety of ESC-derived iMSC for the treatment of Primary Ovarian Insufficiency (NCT03877471). The Tongji Hospital in China is evaluating the safe clinical use of intrauterine injected human ESC-derived iMSCs to treat moderate and severe intrauterine adhesion. In addition to safety this study should bring first insights for clinical effectiveness in supporting the regeneration and repair process within the endometrial region (NCT04232592)^1^^,^^2^.

The pioneer study from Cynata Therapeutics Ltd., of iMSCs in the treatment of GvHD (NCT02923375) is the first completed clinical trial for the use of iMSCs (Bloor et al., 2020). The clinical data confirmed the safety and tolerability of their iMSC product. However, further studies on more than 15 probands and for efficacy testing have to be carried out (Bloor et al., 2020). In this study the authors describe the three-staged generation process in detail. In a first step, the iPSCs are banked and expanded. The iPSCs which were used in the study were generated by Cellular Dynamics International, Inc., from mononuclear blood cells of a healthy adult donor using episomal-based non-integrating reprogramming method oriP/EBNA1-based plasmids (Mack et al., 2011; Bloor et al., 2020). Subsequently, the iPSCs are differentiated into iMSCs in the second step. The final step is the iMSCs expansion and the implementation of the final medicinal product. With this process, according to the authors’ calculations, it is possible to derive 9 × 10^4^ vials, each containing 1 × 10^6^ iPSCs which in the end iMSCs stage have reached a number of 2.9 × 10^15^ iMSCs which represent 29 million therapeutic doses of 1 × 10^8^ cells- all started with iPSC generation from one single blood donation (Bloor et al., 2020). iPSC quality control includes PCR, comparative genomic hybridization and single-nucleotide polymorphism analysis. Since the middle stage the iPSCs are tumorigenic by nature the manufacturer has to ensure the complete absence of undifferentiated iPSCs in the end stage of iMSCs. Therefore, several screening steps are carried out which include medium selection which does not support iPSC growth, physical separation of undifferentiated cell clusters and culture conditions that will favor iMSC growth such as plastic adherence. Furthermore, qPCR and *in vitro* tumorigenicity assays were done. This process is verified by using undifferentiated iPSCs which led to the result that no iPSC colony was detectable thereafter. The iMSCs were characterized by cell surface marker expression CD105+, CD73+, CD90+, CD43/45−, CD31−, and HLA-DR−. A high degree of inter-LOT consistency was claimed based on transcriptome analysis (Bloor et al., 2020). Based on the maximal used dose of 2 × 10^8^ cells per kg body weight which were administered intravenously on Day 0 and Day 7, the authors calculated that a single iPSC Bank would be able to produce 29 million clinical doses of the iMSC product (Bloor et al., 2020). These production capacities are of course highly connected to the capacities of the iPSC bank. By using the iPSC to iMSCs approach the cellular expansion can be shifted from the MSC to the iPSC stage and enable expansion to large quantities with the same starting material (Ozay et al., 2019). The iMSC product has already been applied in other diseases on a pre-clinical level such as asthma, limb ischemia, or rejection of organs (Bloor et al., 2020). The route via PSCs makes those cells also eligible for gene editing technologies such as CRISPR/Cas9 and gene-directed enzyme prodrug therapy (Doudna and Charpentier, 2014).

#### General Outlook

iMSCs as an off-the shelf product with high batch to batch consistency will result in more robust clinical measures (Wilson et al., 2019). Furthermore, iMSCs could serve as a reference material in the research environment to ensure MSC as well as iMSCs standards worldwide. Cell based reference materials (reference cell line) which are available to all laboratories could help in optimizing clinical outcomes and mastering MSC-associated (heterogeneity) challenges (Viswanathan et al., 2014). Manufacturing of such a line is challenging by having large quantities of cells, and stably expandable cells with MSC properties, iMSCs may be of use for this case (Tanavde et al., 2015). Until the use of iMSCs becomes routine in clinical studies critical issues such as optimized generation protocols, deciphering their mode of action and differentiation routes have to be fully understood to ensure higher degree of clinical efficiency and safe use (Zhao and Ikeya, 2018).

## Discussion

In this study on the heterogeneity of MSCs, we comparatively characterized the transcriptomes of MSC subpopulations and the relationships amongst them. We found large commonalities between them but also differences related to the tissue source of the MSCs. In a hierarchical clustering analysis we could stratify the analyzed stem cells into three meta-groups containing PSCs (embryonal and induced), MSCs of adult origin of various tissues and MSCs of young origin (fetal, UC, and iMSCs). Comparing the expressed genes (detection-*p*-value < 0.05) between iMSCs fetal, MSCs, UC-MSCs, BM-MSCs, and AT-MSCs we found most genes (*n* = 9966) overlapping with smaller specific subsets of exclusively expressed genes (n between 29 and 558). Over-represented GOs in the exclusive subsets related to developmental processes in the young MSCs, to adipose-tissue-related (vascular, muscle, and neuronal development) in AT-MSCs and bone-related (skeletal system and limb development) in BM-MSCs. We extended the comparison to MSCs of other distinct sources (AF-MSCs, UdRPCs, and HSCs) generated on different technical platforms and again found the most genes in the overlap (*n* = 6637) and fewer genes in the exclusive subsets (between 301 and 2,280). Here, over-represented GOs in the exclusive subsets related to developmental processes in the AF-MSCs which may be assigned to the young meta-group, over-represented GO terms in UdRPCs included lipid transport and detoxification pointing at renal properties and over-represented GO terms in HSCs included metabolic, cristae, and mitochondrial processes characterizing liver.

We further investigated if we could use the iMSC concept to revert adult cells from heterogeneous populations to a defined MSC initial state and found evidence for that in overlapping developmental biological processes (GOs) with the other young MSCs (fetal and UC-MSCs) and by clustering with the other young MSCs in heatmaps using our previous aging-rejuvenation gene signature (Spitzhorn et al., 2019). These analyses showed that gene-regulatory networks driving rejuvenation are activated in iMSCs. To refine this search to epigenetic causes we performed hierarchical clustering with a gene signature related to epigenetic mechanisms described by Avgustinova and Benitah (2016) and also with this signature we could get defined clusters of PSCs, adult MSCs and marginally fragmented young MSCs. Although this is no direct epigenetic analysis it demonstrates that epigenetic mechanisms connected to the expression of this gene signature are active in the differentiation and rejuvenation of these MSCs. The causal relationship between aging and epigenetic changes has been demonstrated by Horvath’s epigenetic clock enabling prediction of the age based on an individual’s methylation profile (Horvath, 2013). While the aging-related long-term changes manifest more prominently on the methylome but on the other hand work through effects on the transcriptome we could also identify a rejuvenation signature in MSCs on the transcriptome level (Spitzhorn et al., 2019). Distinct lineages and states of cellular differentiation alongside aging are associated with pronounced epigenetics programs in regulating MSC homeostasis (Sui et al., 2020).

**FIGURE 7:**
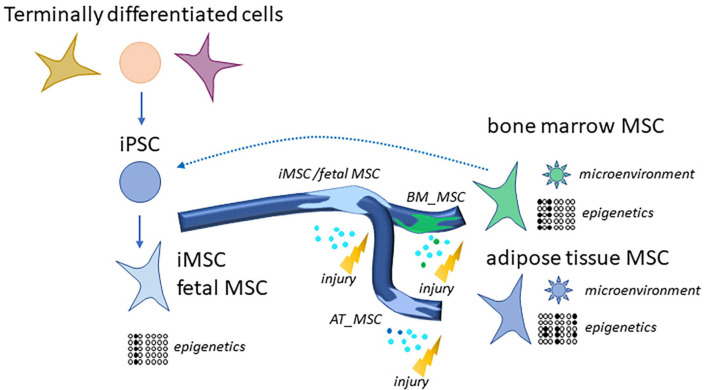
Generation of rejuvenated and more homogenous iMSCs via reprogramming of MSCs or terminally differentiated cells to iPSCs. Distinct MSC subpopulations such as bone marrow MSCs and adipose tissue MSCs have high levels of heterogeneity due to their microenvironments and epigenetic programs. This gets more similar as well as rejuvenated in iMSCs. MSCs as well as iMSCs home into sites of injury and exert beneficial effects which are predominantly due to paracrine signaling and are even superior for the iMSCs as several studies demonstrated.

We propose that the source of the MSCs characterizes the subpopulation by exchanging signals with the microenvironment and by epigenetic programs. We found that iMSCs derived from iPSCs resemble other MSCs of young age such as fetal MSCs and UC-MSCs and are determined by developmental biological processes. That confirms our earlier findings (Spitzhorn et al., 2019) and may imply that the epigenetic programs of MSCs in differentiated and aged microenvironments such as bone marrow or adipose tissue can be reset to an initial state as shown in Figure 7. Also Frobel et al. (2014) report an epigenetic rejuvenation of age- and tissue-related DNA methylation in iPSC-derived MSCs while retaining donor-specific methylation patterns. This initial epigenetic state represented in the iMSCs could provide a means of overcoming heterogeneity and thus a reproducible standard for therapies.

An advantage of MSCs is their more differentiated state compared to PSCs what was considered to protect them from tumorigenicity (Diederichs and Tuan, 2014). However, in recent literature the image of a double-edged sword came up meaning that MSCs can promote and inhibit cancer (Lee and Hong, 2017; Galland and Stamenkovic, 2020). In that context CSCs which are defined rather by CD133 than MSC marker expression have been described to arise from epithelial-mesenchymal-transition (EMT) of cancer cells (Shibue and Weinberg, 2017). This suggests plasticity of these mesenchymal cell types and may raise questions by which changes to the microenvironment and epigenetics they are transformable into each other. Transitions between these cell states may putatively be connected to cancerogenesis or on the other hand have the potential to drive cancer cells to a differentiated state. Thus, their study may elucidate if and in what microenvironment MSCs have tumor-promoting or -inhibiting effects (Diederichs and Tuan, 2014; Papaccio et al., 2017; Galland and Stamenkovic, 2020) with the aim to find the best therapeutic states.

In conclusion, in this review and meta-analysis comparing MSCs from multiple tissues and donor ages we have found predominant commonalities but also differences contributing to heterogeneity. Differences between subpopulations were related to the tissue source environment which also holds for MSC cells such as UdRPCs showing renal properties and HSCs showing hepatic properties but still bearing more commonalities than differences to other MSC types. iMSCs, MSCs derived from iPSCs, have overlapping developmental biological processes with other young MSCs of fetal or umbilical cord origin and thus confirmed our previous rejuvenation signature (Spitzhorn et al., 2019). We demonstrated that rejuvenation can be driven by epigenetic mechanisms and propose that heterogeneity resulting from distinct microenvironmental stimuli and epigenetic patterns may be reset to a rejuvenated state via cellular reprogramming and differentiation into iMSCs.

## Author Contributions

WW, NG, L-SS, and JA wrote the manuscript. WW performed the meta-analysis and follow-up analyses. JA initiated and supervised the study. All authors contributed to the article and approved the submitted version.

## Conflict of Interest

The authors declare that the research was conducted in the absence of any commercial or financial relationships that could be construed as a potential conflict of interest.

## Publisher’s Note

All claims expressed in this article are solely those of the authors and do not necessarily represent those of their affiliated organizations, or those of the publisher, the editors and the reviewers. Any product that may be evaluated in this article, or claim that may be made by its manufacturer, is not guaranteed or endorsed by the publisher.

## Footnotes

1
https://clinicaltrials.gov

2
https://hpscreg.eu/

## Supplementary Material

Supplementary Table Subsets and GO analyses of relevant subsets of the venn diagram of fetal MSCs, iMSCs, adipose-tissue-MSCs, bone-marrow-MSCs and UC-MSCs (Figures 2 and 3). **(A)** subsets of genes, **(B)** Significant GO terms in fetal MSCs (401 genes), **(C)** Significant GO terms in UC-MSCs (558 genes), **(D)** Significant GO terms in iMSCs (374 genes), **(E)** Significant GO terms in adipose-tissue-derived MSCs (29 genes), **(F)** Significant GO terms in bone-marrow-derived MSCs (130 genes), **(G)** Significant GO terms in the intersection of all (9966 genes).

Supplementary Table 2 Subsets and GO analyses of relevant subsets of the venn diagram of amniotic-fluid-derived MSCs (AF-MSCs), urine-derived renal progenitor cells (UdRPCs) and hepatic stellate cells (HSCs) and the common MSC signature determined before (Figure 4). **(A)** subsets of genes, **(B)** Significant GO terms in AF-MSCs (301 genes), **(C)** Significant GO terms in UdRPCs (476 genes), **(D)** Significant GO terms in HSCs (2280 genes).
